# Axial vs equatorial: Capturing the intramolecular charge transfer state geometry in conformational polymorphic crystals of a donor–bridge–acceptor dyad in nanosecond-time-scale

**DOI:** 10.1063/5.0134792

**Published:** 2023-02-06

**Authors:** Krishnayan Basuroy, Jose de J. Velazquez-Garcia, Darina Storozhuk, Robert Henning, David J. Gosztola, Sreevidya Thekku Veedu, Simone Techert

**Affiliations:** 1 Deutsches Elektronen-Synchrotron DESY, Notkestr. 85, 22607 Hamburg, Germany; 2 Center for Advanced Radiation Sources, The University of Chicago, Chicago, Illinois 60637, USA; 3 Center for Nanoscale Materials, Argonne National Laboratory, Lemont, Illinois 60439, USA; 4 Institute of X-ray Physics, University of Göttingen, Friedrich-Hund-Platz 1, 37077 Göttingen, Germany

## Abstract

Two conformational polymorphs of a donor–bridge–acceptor (D-B-A) dyad, *p*-(CH_3_)_2_N-C_6_H_4_-(CH_2_)_2_-(1-pyrenyl)/PyCHDMA, were studied, where the electron donor (D) moiety *p*-(CH_3_)_2_N-C_6_H_4_/DMA is connected through a bridging group (B), –CH_2_–CH_2_–, to the electron acceptor (A) moiety pyrene. Though molecular dyads like PyCHDMA have the potential to change solar energy into electrical current through the process of photoinduced intramolecular charge transfer (ICT), the major challenge is the real-time investigation of the photoinduced ICT process in crystals, necessary to design solid-state optoelectronic materials. The time-correlated single photon counting (TCSPC) measurements with the single crystals showed that the ICT state lifetime of the thermodynamic form, PyCHDMA1 (pyrene and DMA: axial), is ∼3 ns, whereas, for the kinetic form, PyCHDMA20 (pyrene and DMA: equatorial), it is ∼7 ns, while photoexcited with 375 nm radiation. The polymorphic crystals were photo-excited and subsequently probed with a pink Laue x-ray beam in time-resolved x-ray diffraction (TRXRD) measurements. The TRXRD results suggest that in the ICT state, due to electron transfer from the tertiary N-atom in DMA moiety to the bridging group and pyrene moiety, a decreased repulsion between the lone-pair and the bond-pair at N-atom induces planarity in the C–N–(CH_3_)_2_ moiety, in both polymorphs. The Natural Bond Orbital calculations and partial atomic charge analysis by Hirshfeld partitioning also corroborated the same. Although the interfragment charge transfer (IFCT) analysis using the TDDFT results showed that for the charge transfer excitation in both conformers, the electrons were transferred from the DMA moiety to mostly the pyrene moiety, the bridging group has little role to play in that.

## I. INTRODUCTION

Photoinduced charge transfer (PCT) reactions are essential in many biological and chemical reactions of wide significance, such as natural photosynthesis,^1^ repair of DNA lesions,^2^ photoelectric conversion in organic solar cells,^3^ and photodegradation of pollutants^4^ . Especially, the PCT process in natural photosynthesis in plants provides an excellent blueprint for an efficient solar energy conversion procedure that may allow us to produce and store energy in a form useful to us.^5^ The wide impact of the PCT process has propelled the investigation of the same in various model systems to have a detailed understanding of the mechanism, which helped create highly responsive, optoelectronic smart materials for the successful conversion of solar energy into electrical current.^6–10^ The process of solar energy conversion can also be quite useful in replacing our reliance on fossil fuel with an easily available, abundant, inexpensive, eco-friendly renewable energy source.^10^

Often pyrene-based small modeled systems are employed to investigate the PCT process, owing to their high charge carrier mobility,^11,12^ and long-lived singlet excited states.^13^ In these CT molecules, pyrene is usually attached to an electron-rich moiety, such as *N*,*N*′-dimethylaniline (DMA), directly or otherwise, to explore the photoinduced electron transfer (PET) process and the intramolecular CT (ICT) states in different D-A^14–16^ or donor–bridge–acceptor (D-B-A)^17–19^ molecular templates, where pyrene acts as an acceptor (A) and DMA as a donor (D). Though most of the spectroscopic and theoretical studies on pyrene–bridge–DMA systems are focused on analyzing the PCT process in solution,^14,15,17–20^ it is the studies in the crystalline state that deems essential to design suitable solid-state materials.^21^ In this regard, single crystals are considered more efficient in the conversion of photon energies, owing to their lesser defects and grain boundaries.^22^ Despite the obvious gains, structural changes associated with the ICT process in solids are not studied much.^23^

The ICT states can be quite different from the ground state (GS) in terms of electronic structure and molecular geometry, provided the molecule is not very rigid.^6^ Two models—the “twisted” ICT (TICT)^6,24,25^ state with an axial conformation and the “planar” ICT (PICT)^26,27^ state with an equatorial conformation, between the D and A-groups, in the respective ICT state geometries—were proposed. Previously reported, extensive solution-state studies on the ICT process and inter-/intra-molecular exciplex formation dynamics in *N*,*N*′-dimethylaniline-(CH_2_)_n_-(1-pyrenyl) and *N*,*N*′-dimethylaniline-(CH_2_)_n_-(9-anthryl) (where n = 0, 1, 2, 3) series of compounds, did not provide much information about the solid-state conformation of those molecules in the ICT state.^17–19,28–30^ Though pyrene-bridge-DMA systems with flexible (CH_2_)_n_ single-bond connectors germinate uncertainties in the conformation of the molecule, it also provides the potential to have different conformations when single crystals were grown from solutions.^31^ At lower-to-ambient temperatures, the reaction in the solid state still holds the “topochemical postulate” relevant and therefore suggests a minimum of atomic or molecular movement during the solid-state reaction.^32–35^

In recent times, time-resolved x-ray diffraction (TRXRD) or time-resolved photocrystallography has evolved as an effective methodology to do the real-time investigation of photoinduced processes in molecular crystals.^36–40^ The advent of ultrafast lasers, x-ray probe pulses with extreme brilliance, and highly sensitive detectors with fast read-out have facilitated the TRXRD method greatly. Most of the small molecule systems, studied by TRXRD, are designed to investigate light irradiated transient species in spin-crossover systems,^41–43^ purely organic molecules with weak interactions,^44^ metal-to-ligand (MLCT)/ligand-to-metal (LMCT) charge transfer processes,^45,46^ or photoinduced linkage isomerism,^47–49^ in solids. The examples of studying purely organic CT molecules by employing TRXRD are quite rare.^23,50^

**FIG. 1.**
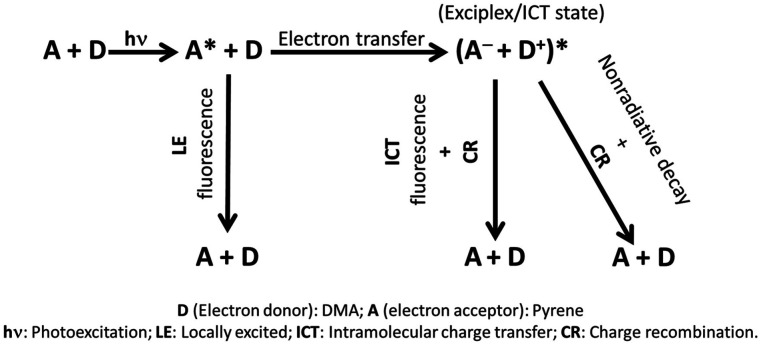
(a) Chemical structure of *p*-(CH_3_)_2_N-C_6_H_4_-(CH_2_)_2_-(1-pyrenyl), PyCHDMA. The torsion angle, α, is defined with bonds in red. (b) and (c) are the molecular conformations of PyCHDMA1 and PyCHDMA20, in crystals, respectively. (d) Superposition of PyCHDMA1 (black) and PyCHDMA20 (red), keeping the pyrene moieties overlapped. (e) and (f) Intermolecular interaction energies between different sets of dimers, calculated using CrystalExplorer, are provided for PyCHDMA1 and PyCHDMA20, respectively (thickness of the cylinders is proportional to the strength of interaction, as the associated values in kcal/mol are suggesting).

In the present study, we have investigated a mono-substituted pyrene derivative, *p*-(CH_3_)_2_N-C_6_H_4_-CH_2_-CH_2_-(1-pyrenyl), PyCHDMA, where electron-rich *p*-(CH_3_)_2_N-C_6_H_4_, *N*,*N*′-dimethylaniline (DMA) that acts as the D is connected to the A, pyrene, through a dimethylene group, –CH_2_–CH_2_– [Fig. 1(a)]. The molecule was exhaustively studied previously in solution to understand the PET process and exciplex formation dynamics with transient absorption and time-resolved fluorescence measurements, but devoid of any solid-state studies.^17–19,28–30^ Despite the obvious capability of capturing the solid-state reactions in real-time, limited penetration of the laser-pump radiation inside the small molecule crystals, restrictions imposed by the geometry of the experiment, and constraints and restraints incorporated by the data collection and processing strategies have made time-resolved photocrystallography measurements quite challenging.^51,52^ Nonetheless, a serendipitous occurrence of two conformational polymorphs^31^ for PyCHDMA has prompted us to delve into the realm of TRXRD measurements to do a comparative analysis of the respective photoinduced ICT geometries, in the crystalline polymorphs.

## II. EXPERIMENTAL SECTION

### A. Synthesis and steady-state spectroscopy

PyCHDMA was synthesized by adopting the procedures mentioned in the literature.^14,30^ The UV–Vis absorption spectra were collected using a Cary-5E UV-VIS spectrophotometer (Varian Australia). The wavelength interval was 0.5 nm, and the path length of the beam inside the cuvettes was 1 mm. The absorption spectra for the compound of interest were corrected using a reference spectrum corresponding to the solvent that is used to dissolve the compound. All the measurements were carried out at RT.

The Jobin Yvon Horiba model Fluorolog 3 FL3 22, equipped with, both, front-face (22°) and right angle (90°) detection, was used to collect the fluorescence emission spectra. The instrument is also equipped with a 450 W Xenon lamp for excitation. All the measurements were performed using 1 mm path-length quartz cuvettes. All the spectra were corrected using the correction files available in the Horiba software that deals with the excitation light intensity and photomultiplier (PMT) response. While collecting the fluorescence emission spectra, the width of the entrance and the exit slit width was 2 nm. The measurements in the solutions were performed after purging with N_2_ gas for 15–20 min.

All the absorption and emission spectra collected in different solvents at different concentrations are shown in Figs. S1 and S2 of the supplementary material.

### B. Single crystal x-ray diffraction

The polymorphs PyCHDMA1 and PyCHDMA20 were grown in an ethyl acetate/ethanol mixture by slow evaporation in two different batches. X-ray data were collected on undulator synchrotron radiation, with λ = 0.620 73 Å at the P11 beamline, in PETRA III, DESY, Hamburg, Germany. Indexing of the x-ray diffraction pattern, unit cell refinement, and spot integration were performed with XDS.^53^ The crystal structures were solved and subsequently refined using the x-ray diffraction datasets collected at 100 K. All the x-ray diffraction datasets were collected in phi scan type mode. The crystal structure was solved using direct methods in SHELXS.^54^ All the structures were refined against F^2^ isotropically, followed by full matrix anisotropic least-squares refinement using SHELXL-97.^55^ For both the structures, all the hydrogen atoms were fixed geometrically, in idealized positions, and allowed to ride with the respective C or N atoms to which each was bonded, in the final cycles of refinement. CCDC deposition numbers for the compounds are 1890 046 (PyCHDMA1) and 1896 006 (PyCHDMA20), which contain the supplementary crystallographic data for this paper, and can be obtained free of charge from the Cambridge Crystallographic Data Center via www.ccdc.cam.ac.uk/data_request/cif. Details of the crystal data and structure refinement parameters are provided in Table S1. The packing of molecules and different intermolecular interactions are shown in great detail in Figs. S3–S8.

### C. Time-correlated single photon counting

**FIG. 2.**
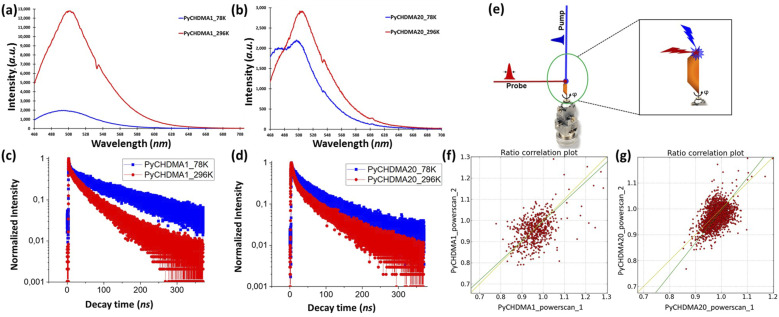
Emission spectra of (a) PyCHDMA1 and (b) PyCHDMA20 at 78 and 296 K (λ_exc_ = 375 nm). Normalized emission decay plots of (c) PyCHDMA1 and (d) PyCHDMA20 at 78 and 296 K (λ_exc_ = 375 nm)). (e) Experimental geometry of the TRXRD measurements. The x-ray probe beam is perpendicular to the laser pump beam. In the inset, it is shown that the laser pump beam is bigger than the x-ray probe beam. Correlation of TR response ratios between datasets powerscan_1 (laser power: 1.0 mJ/mm^2^) and powerscan_2 (laser power: 1.1 mJ/mm^2^) for (f) PyCHDMA1 and (g) PyCHDMA20).

Photoluminescence (PL) spectra and lifetimes in single crystals were measured at the Center for Nanoscale Materials (CNM) at Argonne National Laboratory, using a home-made fluorescence microscope fitted with a liquid nitrogen cooled, continuous flow cryostat (Janis ST-500UC) in CNM. The instrument was based on an Olympus IX-71 inverted microscope. A pulsed 375 nm laser (PicoQuant, DC375M), operated at 4 MHz, was used to excite the sample through a ThorLabs LMU-15X-NUVobjective that was used to both focus the incoming laser light and collect the emitted PL. The collected PL was separated from the exciting laser using a dichroic mirror and a bandpass filter (both Semrock). A low pass filter (LPF) of 468 nm was used for both the PL spectra and TCSPC measurements. The PL was then routed either to a spectrograph (Princeton Instruments, SpectraPro-300) fitted with a CCD camera (Princeton Instruments, PIXIS) or, for lifetime measurements, to a fiber-coupled single photon avalanche diode (SPAD) (Micro Photon Devices, PDM). The output from the SPAD and a trigger pulse from the laser power supply were fed to the two input channels of a time-correlated single photon counting (TCSPC) system (PicoQuant, PicoHarp 300). The relevant lifetimes are provided in Table S4. The emission spectra and normalized emission decay plots are provided in Figs. 2(a)–2(d).

### D. Quantum chemical calculations using DFT (density functional theory) methods

The energy framework analysis was performed using CrystalExplorer 21.5.^56^ For both polymorphs, the intermolecular interaction energies for a 3.8 Å cluster of molecules around the selected central molecule, were calculated at the B3LYP/6-31G (d,p)^57,58^ level of theory, before the energy framework analysis. The framework of energy between molecular pairs is calculated using the geometries obtained from x-ray diffraction measurements, at 100 K, on single crystals, for both polymorphs. The resulting energy frameworks are presented as cylinders joining the centroids of pairs of molecules. The radii of these cylinders are proportional to the magnitude of the intermolecular interaction energies. Frameworks were constructed for electrostatic Coulomb interactions E_ele_ (red cylinders), dispersion interactions E_dis_ (green), and total interaction energies E_tot_ (blue). The intermolecular interaction energies between molecular pairs are calculated by adding the contributions from the electrostatic, polarization, dispersive, and exchange repulsive components, obtained by using unperturbed molecular wave functions, as suggested in the literature [Eq. (1)]:

$$E_{\text{tot}}=k_{\text{ele}}E_{\text{ele}}+k_{\text{pol}}E_{\text{pol}}+k_{\text{dis}}E_{\text{dis}}+k_{\text{rep}}E_{\text{rep}}.$$ (1)

Further inferences drawn, along with the results from the energy framework analysis, are provided in the supplementary material.

Most of the quantum chemical gas phase calculations were performed using density functional theory (DFT) methods at the B3LYP/6-311G** level of theory with the Becke^57^ three-parameter hybrid functional and Lee–Yang–Parr’s^58^ gradient-corrected correlation functional (B3LYP) implemented in the Gaussian16 (G16)^59^ package. A gas-phase potential energy scan (PES) on the torsion angle, α, at 10° intervals, from −180° to 180°, was performed at the M062X/6-311G**^60^ level of theory. The ground state (GS) optimized singlet was used for the calculation of frontier molecular orbitals (MOs) at the occupied ground state and unoccupied virtual state by time-dependent DFT (TDDFT) methods (Fig. S16). TDDFT calculations have also provided theoretical UV–Vis spectra along with the excitation energy of the molecules at the gas phase (Fig. S17). GaussSum 3.0 was used to plot the density of the states diagram (Fig. S18).^61^ GaussView 6.0 was used to plot the frontier orbitals.^62^ Due to the occurrence of many closely lying states within a small energy gap, the density of states (DOS) plot will help us to realize the total concentration of available states within a small given energy range (Fig. S18). Although already provided frontier orbital diagrams are showing that HOMO is mostly occupied by the DMA moiety, whereas the LUMOs are mostly occupied by the pyrene moiety (Fig. S16), the DOS plot provides a clearer and more quantitative picture, with the exact values of energy ranges. The DFT-optimized geometries for the GS and first excited state were obtained at the B3LYP/6-311G** level of theory (Fig. S19).

The results obtained from the TDDFT calculations were further utilized in the analysis and visualization of the charge transfer matrices as heat maps, and for the quantitative analysis of inter- and intra-fragment charge transfer via the IFCT method incorporated in the Multiwfn 3.6 program.^63,64^ The inter-fragment charge transfer (IFCT) method is based on hole–electron analysis and is primarily used for a quantitative assessment of the amount of charge transfer between different fragments, in the process of electron excitation. The IFCT analysis not only provides the amount of inter-fragment charge transfer, but also the atom-to-atom electron/hole transfer in the form of a heat map, known as the charge transfer matrix (CTM) (Figs. S20 and S21). In the present study, the hole and electron distributions were calculated with the IFCT method by employing the Hirshfeld partition. A number of major features for the S_1_ ← S_0_ excitations for both the polymorphs obtained from IFCT analysis are presented here. A detailed analysis of the results is provided in the supplementary material.

The Natural Bond Orbital (NBO) method has been utilized to analyze the intramolecular charge transfer in the polymorphs, especially with a focus on the lone-pair (LP) orbital of nitrogen atom. A strong charge transfer interaction is one where the LP orbital of nitrogen atom works as the donor and the anti-bonding π* orbital of C–C bond in the phenyl moiety of DMA acts as the acceptor. In order to investigate the scope of intermolecular charge transfer between the π–π stacked dimers, NBO calculations were performed using the dimeric geometries obtained from GS single crystal XRD and pink Laue TRXRD measurements. The NBO calculations are carried out in Gaussian16 at M062X/6-311G** level of theory. The outputs from the NBO calculation are viewed using Chemcraft 1.7.18.^65^ Table III, in the main text, lists details of the outcome from the NBO analysis of the polymorphs.

### E. Time-resolved x-ray diffraction (TRXRD) measurements

The time-resolved, pink Laue x-ray diffraction datasets were collected at the 14-ID beamline in the BioCARS station of the Advanced Photon Source (APS; Chicago, Illinois, USA) at the 15 keV undulator setting (pink Laue radiation, ≈0.826 56 Å), within the beamtime available for the standard 24-bunch operation mode (single x-ray pulse length of about 100 ps). The pump–probe time-resolved experiment is designed in such a way that we get the maximum overlap between the pump and the probe on the crystal. The laser-pump beam of 390 nm wavelength is coming perpendicular to the x-ray probe beam [Fig. 2(e)]. All datasets were collected at 100 K upon 38 ps pulses from a Ti:sapphire laser (Spectra-Physics Spitfire Pro laser coupled to a TOPAS optical parametric amplifier), tuned to a wavelength of 390 nm, used as the pump source, with a pump–probe delay of about 1 and 2 ns for both PyCHDMA1 and PyCHDMA20, respectively, but unfortunately, only 1 ns data for PyCHDMA1 and 2 ns datasets for PyCHDMA20 were good enough to proceed. The laser-pump beam has an average cross-section of ∼50 *µ*m, whereas, the x-ray probe beam has an average cross-section of ∼20 *µ*m. The crystals were mounted on glass fibers using Paratone N oil or UHU glue.

It was also important to have the same crystal lattice (same unit cell dimensions) for both, laser-OFF and laser-ON frames, in order to use the RATIO method successfully. To maximize the number of weak reflections observed in all datasets, the pump–probe cycle was repeated 5 to 10 times for each frame before the detector readout. This strategy also helps in statistical background estimation and filtering of the intensities. Frames were collected with an increment of 1°. Laser-ON and laser-OFF frames were recorded in prompt succession to minimize the effect of long-range fluctuations in the beam’s position or intensity. The optimal laser power was selected on the basis of four preliminary short scans; two scans, each with laser power 1.0 and 1.1 mJ/mm^2^, for both PyCHDMA1 and PyCHDMA20, respectively. These short scans have a total angular coverage of 30°. These scans were measured prior to the start of more extensive data collection in order to establish the existence of a response to the laser exposure and the reproducibility of the measurements [Figs. 2(f) and 2(g)]. After going through the short power scans, it was decided to continue the collection of more extensive datasets with a laser power of 1.0 mJ/mm^2^, with 3.8 *µ*J per pulse for all the measurements (Table S11). The datasets were subsequently processed by the RATIO method^66^ (based on the intensity ratios, R = I_ON_/I_OFF_), incorporated in the LaueUtil toolkit.^67,68^ For PyCHDMA1 (2 datasets) and PyCHDMA20 (three datasets), multiple datasets were processed with the LaueUtil toolkit.

The thermal motion increase was modeled by introducing the temperature scale factor (*k*_B_), which relates the atomic displacement parameters, *U*_*ij*_’s, of the laser-ON and laser-OFF structures, in the following way:

$$U_{ij}^{ON}=k_{B}\cdot U_{ij}^{\text{OFF}}.$$

The overall temperature scale parameter *k*_B_ is defined as

$$k_{\text{B}}=\left(B+\Delta B\right)/B,$$

where ΔB is an estimate of the difference between the laser-ON and laser-OFF atomic displacement parameters:

$$k_{\text{B}}=1+\Delta B/8\pi^{2}2\left\langle {\text{U}}_{eq,OFF}\right\rangle .$$

This formalism was found to be a good approximation in the previously studied cases. Similar to the population parameter, *k*_B_ is defined for each dataset independently. The same unit cell was used for both laser-OFF and laser-ON structures, which is reasonable due to the very low-conversion percentage and temperature increase estimated from a plot similar to the Wilson plot and named in the context of pump–probe measurement as the photo-Wilson plot (Figs. S26 and S27).

In a photo-Wilson plot, ln[R_ON/OFF_(**h**)] is plotted against (sin θ/λ)^2^ and then equated to ln[R_ON/OFF_(**h**)] = −2∆Bs^2^(h) + b, where s(h) = sin θ/λ.

Then, from the value of the slope of the ln[R_ON/OFF_(**h**)] vs (sin θ/λ)^2^ plot, we can obtain the value of ∆B.

In order to plot the photodifference and other related maps (photoresidual and photodeformation), all unmerged sets of reflections were scaled, according to the literature procedure, and then merged with SORTAV.^69–71^ In order to obtain photoinduced excited-state (ES) geometry, response-ratio refinement was conducted with the LASER^72,73^ program, which minimizes the following function: $wS_{R}=\sum_{k}w\left(H_{k}\right){\left[R_{o}\left(H_{k}\right)-R_{c}\left(H_{k}\right)\right]}^{2}$, describing discrepancies between the observed and calculated ratios. The calculated ratios are computed as $R_{c}=|F_{c}^{\text{ON}}{|}^{2}/|F_{c}^{\text{OFF}}{|}^{2}$, where $F_{c}^{\text{OFF}}$ is structure factor of the unperturbed (i.e., laser-OFF) structure, and $F_{c}^{ON}$ is the structure factor of the laser-ON structure. The statistical weights were used [i.e., for i-th reflection, *w* = 1/*σ*^2^(R_*o*_)]. Initial atomic coordinates, x, y, and z, and anisotropic displacement parameters (*U*_*ij*_’s) for each atom were taken from the IAM-based refinement. Only the reflections fulfilling the following criterion were considered: σ(*η*_*o*_) ≤ 0.5 (where the response ratio, *η*, defined as *η* = R − 1). The refinement procedure was based on a random spatial distribution (RD) model (disorder-like model) of the excited-state species in a crystal, in which the total calculated structure factor for the laser-ON structure is expressed as $\left|F_{c}^{ON}\right|=P\cdot \left|F_{c}^{ES}\right|+\left(1-P\right).\left|F_{c}^{GS}\right|$, where *P* is the population factor of the species, and *F*_*c*_^*ES*^ and *F*_*c*_^*GS*^ are the ES and GS molecule structure factors, respectively.

A greater detail on data collection, processing, and results from TRXRD measurements is provided in the supplementary material.

## III. RESULTS AND DISCUSSION

### A. Steady-state spectroscopy in solution

**SCHEME 1.**
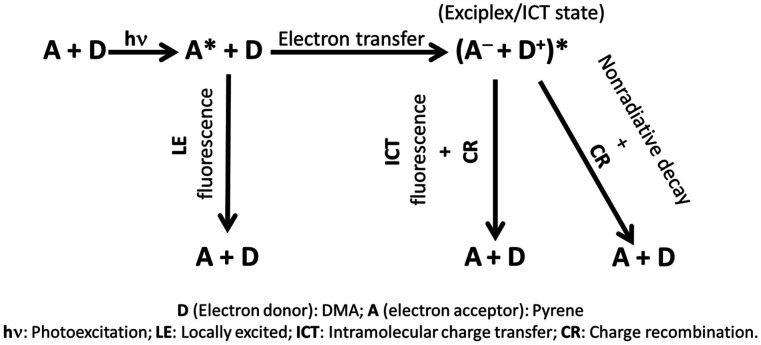
A hypothetical schematic for the generation and relaxation of ICT state in PyCHDMA.

The absorption spectra collected in different solvents (toluene, ethyl acetate, and ethanol) do not show any change in the relative intensity or the position of vibronic bands, with either the changing solvent polarity or the concentration of the solution, suggesting no appreciable interaction between the D and A moieties in the ground state (GS) or any sort of intermolecular electronic coupling in high concentration through *J*- or *H*-aggregation^74^ (Fig. S1). The emission spectra collected at the different solvents show a strong presence of dual fluorescence, both due to the transition from the locally excited (LE) state and the fluorescent exciplex/CT state (Scheme 1; Figs. S1 and S2). While the emission bands, I, III, and IV corresponding to the characteristic pyrene emission from the locally excited (LE) state was clearly visible in toluene and ethyl acetate, in ethanol, along with the other bands, band V at 415 nm also appeared. The broad structureless, high-wavelength CT bands centered at 475 nm (in toluene), 483 nm (in ethyl acetate), and 529 nm (ethanol) clearly show the presence of a bathochromic shift while gradually increasing the polarity of the solvents (Fig. S2). A rapid decrease in the fluorescence yield of the CT band with respect to the LE band with increasing solvent polarity was also observed (Fig. S2). The solvatochromism observed for the CT band maxima in the form of large fluorescence Stokes shift due to the solvation in polar solvents clearly suggests the strong ICT nature of the molecule.^75–78^

PyCHDMA compound is known to form an exciplex between the single excited pyrene, A*, and ground state DMA, D (Scheme 1), through Coulombic attraction.^28–30,79,80^ During the exciplex formation in the excited state, a partial charge transfer occurs from D to A*. The exciplex state, or in this case, the ICT state, can revert back to the ground state through a radiative emission or through the process of charge recombination (CR) by back-electron transfer (BET) (Scheme 1).^81^ Previously reported studies have suggested that the geometry of PyCHDMA does not allow it to take a sandwich conformation with pyrene and DMA in solution, to form an intramolecular exciplex, and, probably, a *cis* or *trans* conformation of the connecting –CH_2_–CH_2_– group puts them at a distance close enough for charge transfer through tunneling.^79,80^

### B. Single crystal x-ray diffraction and energy framework analysis

While set up for growing crystals, PyCHDMA crystallized in two polymorphic forms—PyCHDMA1 (space group P-1) and PyCHDMA20 (space group *P*2_1_/*n*)—in two separate crystallization batches in ethanol/ethyl acetate binary mixtures. Both polymorphs are crystallized in centro-symmetric space groups, with one molecule in the crystallographic asymmetric unit. While, in the crystal structure of PyCHDMA1, pyrene and dimethylaniline are in axial orientations with an interplanar angle of 72.74°, in PyCHDMA20, they are in equatorial conformation with an interplanar angle of 4.28°. The difference in the conformations between the polymorphs can be defined by a torsion about the single bond defined in Fig. 1(a). The respective torsion angle values for PyCHDMA1 and PyCHDMA20 are indicated in Figs. 1(b) and 1(c). The difference in conformation is quite clearly visible in the superposition diagram of PyCHDMA1 and PyCHDMA20 [Fig. 1(d)]. The shortest distance between the N-atom in the DMA moiety and a carbon atom from the pyrene moiety is 7.97 Å, and 8.01 Å, for PyCHDMA1 and PyCHDMA20. The extent of overlap between the π–π stacked pyrene moieties in the PyCHDMA1 and PyCHDMA20 crystals is ∼66% and ∼17%, respectively (Fig. S8), but, the overlap of the effective van der Waals volume^82^ of the π–π stacked molecules is greater in PyCHDMA20 compared to PyCHDMA1. This was also reflected in the energy framework analysis performed with CrystalExplorer 21.5 to understand the stabilization interactions while the molecules pack in crystals. The interaction energies are calculated using the ground state (GS) geometries obtained from single crystal XRD measurements. The interaction energies between the π–π stacked molecular pairs in PyCHDMA1 and PyCHDMA20 crystals are −14.9 and −17.1 kcal/mol, respectively. In both cases, a major contribution to these interaction energies is from dispersion interactions (Table S5).

### C. Quantum chemical calculations with DFT methods

**FIG. 3.**
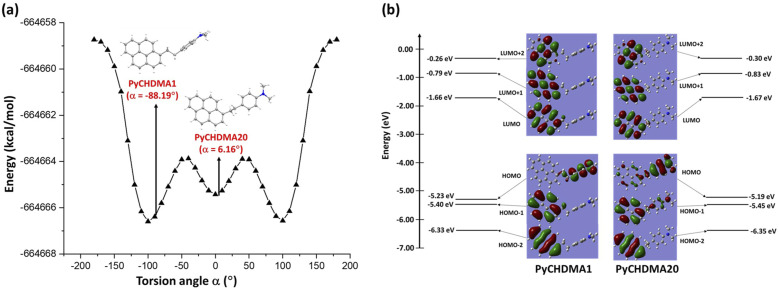
(a) PES plot for energy vs torsion angle. The molecular conformation of the single crystal structures is also shown in the plot. (b) Molecular orbitals (MOs) obtained from TDDFT calculations for PyCHDMA1 and PyCHDMA20 are plotted with energy values.

The difference in the conformations of PyCHDMA1 and PDCHMA20 was further investigated with a gas-phase potential energy scan (PES) of the torsion angle, α, at 10° intervals, from −180° to 180°, at the M062X/6-311G** level of theory [Fig. 3(a)]. The PES results show that the axial conformation between the two π-rings of pyrene and DMA, in PyCHDMA1, is close to a global minimum with respect to the optimized energy values, thus considering the polymorph as representing the stable thermodynamic form,^83^ whereas the equatorial conformation between pyrene and DMA in PyCHDMA20 was close to a local minimum [Fig. 3(a)] and can be considered as the polymorphic representation of the metastable kinetic form.^83^ Figure 3(a) shows that in order to go from the molecular conformation of PyCHDMA1 to the conformation of PyCHDMA20, a potential barrier of height >2 kcal/mol needs to be crossed by rotation about a single bond, suggesting that the nature of the polymorphs is conformational.^31^

The charge transfer characteristics of PyCHDMA1 and PyCHDMA20 monomers with their specific geometry were explored by time-dependent DFT (TDDFT) calculations in the Gaussian16 package at B3LYP/6-311G** level of theory. Mostly, the HOMO was localized at the DMA and LUMO was localized at pyrene, in both the systems [Fig. 3(b)] (Table S6). These spatially separated HOMO (−5.23 eV in PyCHDMA1; −5.19 eV in PyCHDMA20) and LUMO (−1.66 eV in PyCHDMA1; −1.67 eV in PyCHDMA20) induce subtle ICT in both the systems (Table S6).^84^ The formation of ICT states belonging to the LUMO ← HOMO transition is consistent with the observed solvatochromism and large Stokes shifts.^75–78^ The S_2_ ← S_0_ transition is mixed in nature, and the LUMO ← HOMO-1, which is assigned as the LE transition, contributes ∼90% in both cases (Tables S6 and S7).

### D. Charge transfer matrix and IFCT method

The TDDFT results were further utilized in the analysis and visualization of the atom-to-atom and inter-fragment charge transfer during S_1_ ← S_0_ excitation, as heat maps, known as charge transfer matrix (CTM).^63,64^ The CTMs were plotted, to quantitatively analyze the inter- and intra-fragment charge transfer via the inter-fragment charge transfer (IFCT) method incorporated in the Multiwfn 3.6 program, where natural transition orbitals (NTOs)^85^ were used to assign the electronic transitions.

**FIG. 4.**
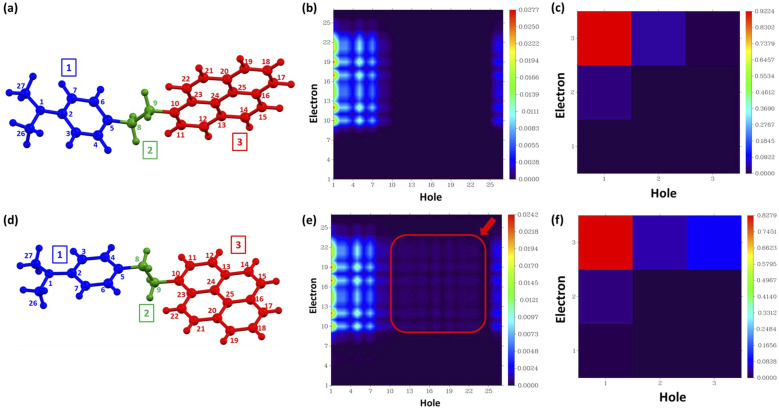
Numbering of atoms and fragments in (a) PyCHDMA1 and (d) PyCHDMA20. The atom-to-atom charge transfer matrix (CTM) of (b) PyCHDMA1 and (e) PyCHDMA20, for S_1_ ← S_0_ excitation, are presented as heat maps. The red square in (e) highlights the fact that the atom-to-atom charge transfer within the pyrene moiety is much more pronounced in PyCHDMA20, compared to PyCHDMA1. The inter-fragment charge transfer (IFCT) matrices of (c) PyCHDMA1 and (f) PyCHDMA20, for S_1_ ← S_0_ excitation, are shown as heat maps.

For the S_i_ ← S_0_ transition, the change in electron density Δρ was defined as

$$\Delta \rho \left(S_{i}\leftarrow S_{0}\right)=\underset{k}{\sum }{\left|\psi_{ik}\left(virtual\right)\right|}^{2}-\underset{k}{\sum }{\left|\psi_{ik}\left(occupied\right)\right|}^{2},$$

where *ψ*_ik_(occupied) and *ψ*_ik_(virtual) are the NTO pairs for the transition. The hole and electron distributions were calculated by employing the Hirshfeld partition. The inter-and intra-fragment electron transfer was studied by dividing the entire molecule into three fragments—pyrene (1st fragment), –CH_2_–CH_2_– (second fragment), and DMA (third fragment) [Figs. 4(a) and 4(d)]. The contributions of various fragments to holes and electrons, for S_1_ ← S_0_ transitions, are also presented (Table S8). For the single electron excitation, the electron on the DMA moiety is reduced by 0.9510 during the electron excitation process, while the pyrene moiety has gained 0.9626 electrons (Table I). Similarly, for PyCHDMA20 fragment 1, the DMA moiety lost 0.8527 electrons and pyrene moiety gained 0.8701 electrons, during the S_1_ ← S_0_ excitation process (Table I). The CTMs in Figs. 4(b) and 4(e), which represent atom-to-atom charge transfer, show that atom no. 1 (N-atom) in the DMA moiety is transferring most electrons to atom nos. 10, 12, 17, and 19, in the pyrene moiety, for both the conformers. Figure 4(e) also shows that the redistribution of electrons within the pyrene moiety is more for PyCHDMA20 compared to PyCHDMA1, as highlighted with a red square on the map (Fig. 4) (Table I). Figures 4(c) and 4(f), which represent the inter-fragment CTMs for both conformers, suggest that, mostly electron is transferred from DMA (first fragment) to pyrene (third fragment), for the S_1_ ← S_0_ excitation. The bridging group, –CH_2_–CH_2_–, has very little contribution to the electron transfer from DMA to pyrene.

**TABLE I.** Interfragment and intrafragment electron transfer in PyCDMA1 and PyCHDMA20 for S_1_ ← S_0_ transition, calculated with the IFCT method in Multiwfn. Boldface denotes the amount of electron transfer between the fragments.

Inter-fragments		Net	Intra-fragment	Inter-fragments		Net	Intra-fragment
PyCHDMA1				PyCHDMA20			
1 → 2	1 ← 2	1 → 2	1 ↔ 1	1 → 2	1 ← 2	1 → 2	1 ↔ 1
**0.02871**	**0.00009**	**0.02862**	**0.00213**	**0.02559**	**0.00026**	**0.02533**	**0.00474**
1 → 3	1 ← 3	1 → 3	2 ↔ 2	1 → 3	1 ← 3	1 → 3	2 ↔ 2
**0.92238**	**0.00001**	**0.92237**	**0.00126**	**0.82786**	**0.00052**	**0.82733**	**0.00141**
2 → 3	2 ← 3	2 → 3	3 ↔ 3	2 → 3	2 ← 3	2 → 3	3 ↔ 3
**0.04041**	**0.00015**	**0.04026**	**0.00486**	**0.04559**	**0.00282**	**0.04277**	**0.09121**

### E. Geometry optimization using the DFT method

The ground state (GS) and 1st excited state (ES) geometries of both conformers were optimized at B3LYP/6-311G** level of theory. The major difference between the GS and first ES optimized geometry was reflected in the movement of the tertiary N-atom in the DMA moiety, which involves N1 going into the plane containing three carbon atoms directly bonded to it and the shortening of the N1-C2 bond (Fig. S19). The pyramidalization of the tertiary amine in the DMA moiety is due to the presence of a lone pair on the N atom, which heavily contributes to the ICT, as exhibited earlier with IFCT methods for S_1_ ← S_0_ excitation. As a result, the pyramidalization goes down in the ICT state. This pyramidal-to-planar structural change in the course of photoinduced electron transfer was reported earlier for the pyrene-tri-n-butylamine (TBA) compound.^86,87^ The reduction in the pyramidalization was quite similar for the first ES optimized geometries, in both the conformers (The N atom moved into the plane by 0.171 Å for PyCHDMA1 and 0.173 Å for PyCHDMA20) (Fig. S19).

### F. Time-correlated single photon counting (TCSPC)

Apart from the axial/equatorial orientation between the pyrene and DMA moieties, the overall differences in the intermolecular interactions or stacking behavior in crystals, for the conformers, are reflected in the TCSPC results while exciting with the 375 nm laser at 78 and 296 K (Table S4). A 468 nm LP filter was used to get rid of any emission below that wavelength, since the exciplex/ICT band or excimer band was mostly appearing beyond that range in the solution (Figs. 2 and S1). For both the crystals, the emission decay profile is bi-exponential at 296 and 78 K [Table S4 and Figs. 2(c) and 2(d)]. The comparatively longer lifetimes observed for both the crystals can be attributed to the excimer lifetime that has formed while exciting with 375 nm radiation. The shorter lifetimes—∼3 ns for PyCHDMA1 and ∼7 ns for PyCHDMA20—can be attributed to the emissive exciplex/ICT state. The lifetimes of the ICT states in crystals are in good agreement with earlier reported studies.^23,88^ The excimer population for PyCHDMA20 goes significantly up from 296 K (18%) to 78 K (43%), which also became evident with the appearance of a second shoulder around 480 nm [Fig. 2(b)].

### G. Time resolved x-ray diffraction (TRXRD)

The changes in the geometry following the photoexcitation were captured in great detail by the pump–probe, time-resolved x-ray diffraction (TRXRD) measurements in the pink Laue regime. To capture the ICT state geometries of PyCHDMA1 and PyCHDMA20 molecules, in crystals, a laser pump radiation of 390 nm was decided for use for both polymorphic crystals, since the TDDFT results suggest that the energy of the HOMO–LUMO transition, which is of ICT nature, lies close to that range (Table S7). The crystals were pumped with 390 nm radiation and subsequently probed by the ≈100 ps x-ray pulse (at 15 keV), with a pump−probe delay of 1 and 2 ns for the PyCHDMA1 and PyCHDMA20 datasets, respectively, since the ICT state lifetimes in both the crystals are longer than 3 ns, as suggested by the TCSPC results (Table S4). In fact, the packing of π–π stacked dimers suggests that there could be *J*-aggregation, which would further bathochromically shift the absorption bands for crystals (Fig. S8). Moreover, we always intend to catch the tail of the absorption band, and not the maximum, in order to avoid large absorption of photons at the surface and consequently a reduced laser penetration.

**FIG. 5.**
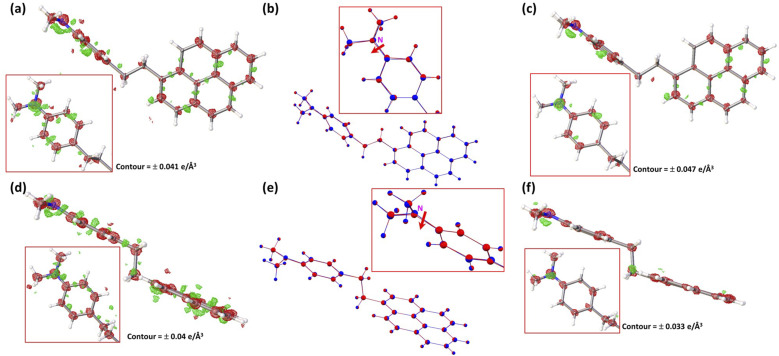
Photodifference maps of (a) PyCHDMA1 and (d) PyCHDMA20 (Green is positive density and red is negative density). Superposition of the GS and ES refined geometries for (b) PyCHDMA1 and (e) PyCHDMA20 (Blue is for GS structure and red for ES structure). The contours of the maps are also indicated in the figures. Photodeformation maps of (c) PyCHDMA1 and (f) PyCHDMA20. Maps were drawn using the combined scale-merged datasets.

Before the beginning of extensive dataset collection, the existence of a reproducible photo-induced response in the crystals was verified by collecting a couple of short datasets with varied laser power [Figs. 2(f) and 2(g)].^89^ The different datasets collected with different crystals with the same pump–probe delay were jointly refined against a common excited state (ES) geometry model (Table II). Subsequently, a combined scale-merged dataset was obtained by scaling those well-correlated individual datasets by a scale factor k(*η*)_set_ = ⟨|*η*|⟩_all_/⟨|*η*|⟩_set_, in which ⟨|*η*|⟩_all_ is the average response ratio, defined as *η*_ON/OFF_(h) = R_ON/OFF_(h) − 1, over all measured reflections and ⟨|*η*|⟩_set_ is the average over all the reflections measured in a particular dataset.^90^ The combined scale datasets were then created to plot photodifference maps,^90^ that reliably illustrate the laser-induced differences between the laser-ON (ES) and laser-OFF (GS) electron density distribution.^91,92^ The photodifference maps suggest a strong shift of the tertiary N-atom into the plane containing three carbon atoms bonded to it [Figs. 5(a) and 5(d)], quite similar to what was obtained with the theoretically optimized geometries of the first excited state, with DFT methods.

**TABLE II.** Data processing parameters, and population and temperature scale factors obtained from individual and combined datasets after LASER refinement.

Datasets	Nref^a^	C(%)^b^	P(%)^c^	*k*_B_	*k*_B_ from photo-Wilson plots
PyCHDMA1_1 ns	1620	51.8	1.30(11)	1.055(2)	1.075
PyCHDMA1_1ns1	1310	42.1	1.05(8)	1.046(2)	1.065
PyCHDMA20_2 ns	2396	38.2	1.59(12)	1.058(1)	1.072
PyCHDMA20_2ns1	1997	31.8	1.58(16)	1.042(2)	1.085
PyCHDMA20_2ns2	2184	34.8	0.89(10)	1.057(1)	1.054

^a^ Number of reflections, after merging in SORTAV.

^b^ Completeness of data.

^c^ Population of excited state species. The completeness for all the datasets was calculated at 0.59 Å^−1^ resolution.

The observations from photodifference maps helped in selecting parameters in intensity-ratio (R = I_ON_/I_OFF_)-based least-squares refinements with the LASER program.^72,73^ The thermal motion increase was modeled by introducing the temperature scale factor, *k*_B_ = *U*_*ij*_^ON^/*U*_*ij*_^OFF^, which relates the *U*_*ij*_’s of the laser-ON and laser-OFF structures. The *k*_B_ values obtained from the LASER refinement results are not far off from the values obtained from the slope of the photo-Wilson plots^93^ for both the conformers (Table II).

For PyCHDMA1, the excited state (ES) population of the two datasets is 1.30% and 1.05%, whereas for PyCHDMA20, the ES population ranges from 0.89% to 1.59% (Table II). The relatively low population of ES species and the small increment of the Debye–Waller factor (B-factor) can be attributed to the fact that the operating power of the pump laser was kept to a level at which sufficient amount of data could be collected without damaging the crystal and probably to the low quantum efficiency at 100 K, as suggested, due to the reduction in the emission maxima corresponding to the ICT band [Figs. 2(a) and 2(b)] with lowering the temperature, as suggested elsewhere too.^23,88^ A lower completeness of the datasets can be attributed to the fact that all of them were collected in *φ*-scan mode, and a portion (10%–30%) of the reflections remains un-indexed while processing with the RATIO method, due to change in the unit cell dimension for heating. This is why the final processed data have a very low *k*_B_ value, which is quite suitable for proceeding to the next step for the refinement of the excited state geometry.

The refined excited state geometry shows a reduction of the bond length N1-C2 in DMA moieties for both the conformers by 0.04 Å in PyCHDMA1 and 0.02 Å in PyCHDMA20, apart from other bond length changes (Tables S14 and S16). The result of intramolecular electron transfer in ES is quite clear in the form of shortening of the N-C bond and a reduction in the pyramidality at the tertiary N-atom within the DMA moiety by 0.114 and 0.087 Å in PyCHDMA1 and PyCHDMA20, respectively [Figs. 5(b) and 5(e)]. Except for the drastic movement of tertiary N-atom into the plane containing three carbon atoms—all bonded to it—the extent of atomic shifts is somewhat less pronounced with the DFT optimized 1st excited state geometries compared to all the TRXRD results (Tables 14–S17). The torsion angle for the rotation about the bond N1-C2, which is not very different between the conformers, does not show any significant change (Table S20; Fig. S29). The movement of the pyrene moiety as a rigid body, following the photoexcitation, is not significant either in terms of rotation or translation and probably in a way validates the topochemical postulate at low temperature in solids (Table S21).

Following the ES model refinement by the LASER program, the photodeformation maps,^46,92^ were calculated using the refined model parameters of ES, which present the difference between the densities calculated with the ES parameters and the GS structure [Figs. 5(c) and 5(f)]. Both, photodifference maps and the photodeformation maps, show a pronounced atomic shift at the N-atom position for both the conformers (Fig. 5). This could be because not only does the N-atom have one more electron than the C-atoms bonded to it, but it also moves more than the rest of them, in the ES due to its loss of lone-pairs in the process of PET (Table S30). The atomic shifts are slightly more pronounced for PyCHDMA1 datasets than PyCHDMA20 datasets (Table S30). The reason could be that the TRXRD datasets presented here are collected at different pump–probe delays.

### H. Natural bonding orbital (NBO) calculation with GS and ES crystal structures

In order to know the ICT interaction energies involving the DMA moiety, especially the N-atom, natural bond orbital (NBO)^94^ analysis at M06-2X/6-311G** level of theory in the gas phase, using the GS and ES geometries, from x-ray diffraction studies and DFT optimization, was utilized. The NBO analysis allowed us to calculate the donor–acceptor interaction energies involving the lone pair (LP) of N-atom as the donor, from 2nd order perturbation theory analysis of the Fock matrix in NBO basis, with E(2) > 0.05 kcal/mol, as listed in Tables S22–S25. The interaction energies provide a measure of the strength of the intramolecular charge transfer interaction. The reduced lone-pair feature at the N atom of the DMA moiety in the ES geometry, obtained from single crystal XRD studies for both the polymorphs, is established using the NBO calculations.^95^ Table III suggests an increased p character (ES: 99.34% in PyCHDMA1 and 98.98% in PyCHDMA20) of the lone-pair (LP) orbital in the ES for both the polymorphs, validating the prominent sp2 character for the N-atom in ES refined geometry. Furthermore, it shows a stronger overlap between LP and anti-bonding π* C–C orbitals at ES, with higher stabilization energy E(2) values (Fig. S30; Table III). However, in both the conformers, the GS shows a slightly weaker overlap between LP and π* C–C orbitals compared to ES due to the mixture of s and p characters in the LP of N-atom. Therefore, as a result of larger intramolecular charge transfer in ES, the N–C bond gets shortened, and the repulsion between the lone pair and the bond pair at the tertiary N-atom decreases, inducing the planarity in the C–N–(CH_3_)_2_ moiety (Fig. S29).

**TABLE III.** The strongest intramolecular charge transfer interactions involving the donor LP orbital of the N-atom and acceptor π* C–C orbitals, in PyCHDMA1 and PyCHDMA20 geometries obtained from single crystal XRD studies, based on the 2nd order perturbation theory analysis of Fock matrix in NBO basis, along with the hybridization, are listed. [Note: E(*i*) corresponds to Lewis type “filled” donor orbitals. E(*j*) corresponds to non-Lewis type “unfilled” acceptor orbitals. BD = 2-center bond, BD* = 2-center anti-bonds. For each donor NBO (*i*) and acceptor NBO (*j*), the stabilization energy *E*(2) associated with *i* → *j* delocalization is determined as $E\left(2\right)=\Delta E_{ij}^{\left(2\right)}=\frac{q_{i}F{\left(i,j\right)}^{2}}{\varepsilon_{j}-\varepsilon_{i}}$, where *q*_i_ is the donor orbital occupancy. *ε*_i_ and *ε*_j_ are diagonal elements (orbital energies) of the NBO Fock matrix. *F*(*i*,*j*) is the off-diagonal NBO Fock matrix elements.]

Donor NBO’s (i)/hybridization	Acceptor NBO’s (j)/hybridization	E(2) (kcal/mol)	E(j) − E(i) (a.u.)	F(i, j) (a.u.)
PyCHDMA1 (GS)				
93. LP (1) N1 s (4.59%) p20.77 (95.39%) d0.00 (0.02%)	565. BD*(2) C2 – C10	45.66	0.37	0.12
	(54.62%) C2 s(0.02%) p99.99(99.94%)			
	d1.91(0.04%)			
	(45.38%) C10 s(0.00%) p1.00(99.97%)			
	d0.00(0.02%)			
PyCHDMA1 (ES)				
93. LP (1) N1 s (0.65%) p99.99 (99.34%) d0.01 (0.01%)	571. BD*(2) C10 - C18	63.65	0.33	0.14
	(56.30%) C10 s(0.36%) p99.99(99.60%)			
	d0.09(0.03%)			
	(43.70%) C18 s(0.80%) p99.99(99.18%)			
	d0.02(0.02%)			
PyCHDMA20 (GS)				
93. LP (1) N1 s (4.05%) p23.66 (95.93%) d0.00 (0.02%)	564. BD*(2) C2 - C3	47.94	0.37	0.12
	(54.66%) C2 s(0.03%) p99.99(99.94%)			
	d1.31(0.04%)			
	(45.34%) C3 s(0.00%) p1.00(99.98%)			
	d0.00(0.02%)			
PyCHDMA20 (ES)				
93. LP (1) N1 s (1.03%) p96.39 (98.97%) d0.01 (0.01%)	571. BD*(2) C10 - C18	55.34	0.36	0.13
	(54.91%) C10 s(0.01%) p1.00(99.95%)			
	d0.00(0.04%)			
	(45.09%) C18 s(0.00%) p1.00(99.97%)			
	d0.00(0.03%)			

NBO analyses for the interaction energies between the π–π stacked dimers were also performed at M06-2X/6-311G** level of theory in the gas phase, using the GS and ES geometries obtained by x-ray diffraction studies.^96^ For PyCHDMA1, the interaction energies between the π–π stacked dimers are higher in ES compared to GS by 1.43 kcal/mol (Table S26), but no such differences are observed for PyCHDMA20 (Table S27). This could be attributed to the fact that the pyrene moiety as a rigid body rotates more in PyCHDMA1 from GS to ES, compared to PyCHDMA20 (Table S21). Though the intermolecular interactions between the π–π stacked dimers are not contributing much to the ES CT process, the interaction energies suggest dimerization for both the conformers at GS and ES.^95^ The results from the output of NBO analyses in Gaussian also provide the individual atomic charges calculated by natural population analysis (NPA) and Hirshfeld population analysis (HPA) (Tables S30–S32). Both the analyses show that the negative charge on the N-atom has reduced in the ES for both the polymorphs. The analyses also showed that the reduction in negative charge on the DMA moiety and the gain of negative charge on the connecting –CH_2_–CH_2_– group and the pyrene moiety is more for PyCHDMA20 compared to PyCHDMA1. The partial atomic charge analysis also showed that, contrary to what IFCT analysis suggested, the bridging group also played a crucial role in the ICT for both conformers.

## IV. CONCLUSIONS

The ICT state geometry for the conformational polymorphs of a purely organic D–B–A dyad in single crystals has never been captured by TRXRD or time-resolved photocrystallography. The results are particularly encouraging, since both the crystals were photoexcited with 390 nm radiation, which falls within the visible spectral range (∼380–740 nm) of the incoming solar energy, which comprises roughly 43% of its total contribution. Though, purely organic molecular dyads such as PyCHDMA have the potential to change solar energy into electrical current through the process of photoinduced ICT, the major challenge that remains is the difficulties associated with the real-time investigation of the process in crystals, in *ns* time-scale. Even with good diffraction quality crystals, the sample may lack in the sheer number of crystals and homogeneity in their size or shape, otherwise required, in order to be tried for serial femtosecond crystallography methods. The asterism with pink Laue or Laue sources can also make the diffraction spot shapes too streaky to process reliably in the TRXRD method.

In the present study, the IFCT analysis using the TDDFT results has shown, in both conformers, that the electron transfer happens probably through a tunneling mechanism, due to the proximity of the D and A, and that the bridging group did not have much role in that—this could be because of the high energy splitting between the relevant states of the donor and bridge (∆E_DB_),^97^ as analyzed by the IFCT method—whereas the partial atomic charge analysis using the GS x-ray diffraction and TRXRD results suggest that while the electron transfer has happened from DMA to pyrene, the bridging group has also received a fair amount of negative charge in the process. There is no significant difference in the pattern of atomic shifts between the conformers while capturing the ICT state geometries with TRXRD measurements with ∼100 ps temporal resolution. The NPA analysis and the partial atomic charge analysis with Hirshfeld partitioning suggested that more electrons are transferred from the DMA moiety, in PyCHDMA20 crystals compared to PyCHDMA1. This difference could be due to either a different delay time of probing or the conformational difference between the dyads. The TRXRD results suggest that for both conformers, a significant amount of intramolecular electron transfer from the N-atom in the DMA moiety to the bridging group and pyrene moiety had taken place, and the shortening of the N1-C2 bond and a reduction of pyramidalization at the same N-atom, at ES, exhibits that clearly. The transfer of electrons from the N-atom also reduced the repulsion between the lone-pair and the bond-pair at the same tertiary N-atom, which, in turn, induced planarity in the C–N–(CH_3_)_2_ moiety. Moreover, the difference between the IFCT and TRXRD, as well as the partial atomic charge analysis results, regarding the role played by the bridging group in the PET process, could be attributed to the fact that in TRXRD, we are treating the pyrene moiety as a rigid body in a single crystal environment and not refining the individual atomic positions within the same, whereas the IFCT analysis is based on the TDDFT calculation of the molecule in gas-phase with optimized geometry in the GS.

## SUPPLEMENTARY MATERIAL

See the supplementary material for UV–Vis absorption, fluorescence emission spectra in solution, crystal data and structure refinement parameters, conformational analysis of the molecular structures, intermolecular hydrogen bond parameters, packing of molecules in single crystals, TCSPC results in single crystals, results from IFCT analyses, results of theoretical calculations by DFT methods, TRXRD data collection, processing, and results, and NBO calculation results.

## Data Availability

The data that support the findings of this study are available within the article and its supplementary material. Additional data are available from the corresponding author upon reasonable request.

## References

[1] J. Deisenhofer and H. Michel, Science 245, 1463 (1989). 10.1126/science.245.4925.1463 17776797

[2] D. B. Hall, R. E. Holmlin, and J. K. Barton, Nature 382, 731 (1996). 10.1038/382731a0 8751447

[3] P. Song, Y. Li, F. Ma, T. Pullerits, and M. Sun, Chem. Rec. 16, 734 (2016). 10.1002/tcr.201500244 26853631

[4] K. Ye, Y. Li, H. Yang, M. Li, Y. Huang, S. Zhang, and H. Ji, Appl. Catal. B 259, 118085 (2019). 10.1016/j.apcatb.2019.118085

[5] O. Kruse, J. Rupprecht, J. H. Mussgnug, G. C. Dismukes, and B. Hankamer, Photochem. Photobiol. Sci. 4, 957 (2005). 10.1039/b506923h 16307108

[6] Z. R. Grabowski, K. Rotkiewicz, and W. Rettig, Chem. Rev. 103, 3899 (2003). 10.1021/cr940745l 14531716

[7] Z. Shuai and Q. Peng, Phys. Rep. 537, 123 (2014). 10.1016/j.physrep.2013.12.002

[8] E. J. Piechota and G. J. Meyer, J. Chem. Educ. 96, 2450 (2019). 10.1021/acs.jchemed.9b00489

[9] M. Mrinalini and S. Prasanthkumar, ChemPlusChem 84, 1103 (2019). 10.1002/cplu.201900365 31943959

[10] M. E. El-Khouly, E. El-Mohsnawy, and S. Fukuzumi, J. Photochem. Photobiol. C 31, 36 (2017). 10.1016/j.jphotochemrev.2017.02.001

[11] H.-Y. Oh, C. Lee, and S. Lee, Org. Electron. 10, 163 (2009). 10.1016/j.orgel.2008.10.015

[12] H. Cho, S. Lee, N. S. Cho, G. E. Jabbour, J. Kwak, D.-H. Hwang, and C. Lee, ACS Appl. Mater. Interfaces 5, 3855 (2013). 10.1021/am4005368 23560572

[13] A. Nakajima, Bull. Chem. Soc. Jpn. 46, 2602 (1973). 10.1246/bcsj.46.2602

[14] S. Techert, S. Schmatz, A. Wiessner, and H. Staerk, J. Phys. Chem. A 104, 5700 (2000). 10.1021/jp9935384

[15] S. Techert, A. Wiessner, S. Schmatz, and H. Staerk, J. Phys. Chem. B 105, 7579 (2001). 10.1021/jp004370l

[16] S. Thekku Veedu, D. Raiser, R. Kia, M. Scholz, and S. Techert, J. Phys. Chem. B 118, 3291 (2014). 10.1021/jp4121222 24601820

[17] T. Okada, M. Migita, N. Mataga, Y. Sakata, and S. Misumi, J. Am. Chem. Soc. 103, 4715 (1981). 10.1021/ja00406a009

[18] N. Mataga, S. Nishikawa, T. Asahi, and T. Okada, J. Phys. Chem. 94, 1443 (1990). 10.1021/j100367a045

[19] N. Mataga, H. Chosrowjan, and S. Taniguchi, J. Photochem. Photobiol. C 6, 37 (2005). 10.1016/j.jphotochemrev.2005.02.003

[20] S. Mondal and D. N. Nath, Spectrochim. Acta A 230, 118019 (2020). 10.1016/j.saa.2019.118019 31955115

[21] T. M. Figueira-Duarte and K. Müllen, Chem. Rev. 111, 7260 (2011). 10.1021/cr100428a 21740071

[22] Z. Chu, M. Yang, P. Schulz, D. Wu, X. Ma, E. Seifert, L. Sun, X. Li, K. Zhu, and K. Lai, Nat. Commun. 8, 2230 (2017). 10.1038/s41467-017-02331-4 29263379 PMC5738431

[23] S. Techert and K. A. Zachariasse, J. Am. Chem. Soc. 126, 5593 (2004). 10.1021/ja0379518 15113231

[24] W. Rettig and W. Majenz, Chem. Phys. Lett. 154, 335 (1989). 10.1016/0009-2614(89)85366-7

[25] R. Lapouyade, A. Kuhn, J.-F. Letard, and W. Rettig, Chem. Phys. Lett. 208, 48 (1993). 10.1016/0009-2614(93)80075-z

[26] K. A. Zachariasse, M. Grobys, T. Von der Haar, A. Hebecker, Y. V. Il'ichev, O. Morawski, I. Rückert, and W. Kühnle, J. Photochem. Photobiol A: Chem. 105, 373 (1997). 10.1016/s1010-6030(96)04601-1

[27] K. A. Zachariasse, Chem. Phys. Lett. 320, 8 (2000). 10.1016/s0009-2614(00)00230-x

[28] T. Okada, T. Fujita, M. Kubota, S. Masaki, N. Mataga, R. Ide, Y. Sakata, and S. Misumi, Chem. Phys. Lett. 14, 563 (1972). 10.1016/0009-2614(72)87208-7

[29] T. Okada, T. Saito, N. Mataga, Y. Sakata, and S. Misumi, Bull. Chem. Soc. Jpn. 50, 331 (1977). 10.1246/bcsj.50.331

[30] M. Migita, T. Okada, N. Mataga, Y. Sakata, S. Misumi, N. Nakashima, and K. Yoshihara, Bull. Chem. Soc. Jpn. 54, 3304 (1981). 10.1246/bcsj.54.3304

[31] A. J. Cruz-Cabeza and J. Bernstein, Chem. Rev. 114, 2170 (2014). 10.1021/cr400249d 24350653

[32] M. D. Cohen and G. M. J. Schmidt, J. Chem. Soc. 1964, 1996. 10.1039/JR9640001996

[33] M. D. Cohen, G. M. J. Schmidt, and F. I. Sonntag, J. Chem. Soc. 1964, 2000. 10.1039/jr9640002000

[34] G. M. J. Schmidt, Pure Appl. Chem. 27, 647 (1971). 10.1351/pac197127040647

[35] A. Ravi, S. Z. Hassan, S. Bhandary, and K. M. Sureshan, Angew. Chem., Int. Ed. 61, e202200954 (2022). 10.1002/anie.202200954 35258143

[36] S. Techert, F. Schotte, and M. Wulff, Phys. Rev. Lett. 86, 2030 (2001). 10.1103/physrevlett.86.2030 11289847

[37] C. D. Kim, S. Pillet, G. Wu, W. K. Fullagar, and P. Coppens, Acta Crystallogr., Sect. A: Found. Adv. 58, 133 (2002). 10.1107/s0108767301017986 11832582

[38] P. Coppens, Angew. Chem., Int. Ed. 48, 4280 (2009). 10.1002/anie.200900910 19378302

[39] P. Coppens, J. Phys. Chem. Lett. 2, 616 (2011). 10.1021/jz200050x

[40] L. E. Hatcher and P. R. Raithby, Coord. Chem. Rev. 277–278, 69 (2014). 10.1016/j.ccr.2014.02.021

[41] H. Cailleau, M. Lorenc, L. Guérin, M. Servol, E. Collet, and M. Buron-Le Cointe, Acta Crystallogr., Sect. A: Found. Adv. 66, 189 (2010). 10.1107/s0108767309051046 20164642

[42] J. D. J. Velazquez-Garcia, K. Basuroy, D. Storozhuk, J. Wong, S. Demeshko, F. Meyer, R. Henning, and S. Techert, Dalton Trans. 51, 6036 (2022). 10.1039/d1dt04255f 35352719

[43] J. D. J. Velazquez-Garcia, K. Basuroy, D. Storozhuk, J. Wong, S. Demeshko, F. Meyer, R. Henning, and S. Techert, Dalton Trans. 51, 17558 (2022). 10.1039/d2dt02638d 36315244 PMC9749069

[44] K. Basuroy, Y. Chen, S. Sarkar, J. Benedict, and P. Coppens, Struct. Dyn. 4, 024501 (2017). 10.1063/1.4978240 28382318 PMC5346101

[45] A. Makal, J. Benedict, E. Trzop, J. Sokolow, B. Fournier, Y. Chen, J. A. Kalinowski, T. Graber, R. Henning, and P. Coppens, J. Phys. Chem. A 116, 3359 (2012). 10.1021/jp300313s 22385365 PMC3545449

[46] K. N. Jarzembska, R. Kamiński, B. Fournier, E. Trzop, J. D. Sokolow, R. Henning, Y. Chen, and P. Coppens, Inorg. Chem. 53, 10594 (2014). 10.1021/ic501696y 25238405 PMC4315237

[47] P. Coppens, D. V. Fomitchev, M. D. Carducci, and K. Culp, J. Chem. Soc., Dalton Trans. 1998, 865. 10.1039/A708604K

[48] N. Casaretto, D. Schaniel, P. Alle, E. Wenger, P. Parois, B. Fournier, E.-E. Bendeif, C. Palin, and S. Pillet, Acta Crystallogr., Sect. B: Struct. Sci., Cryst. Eng. Mater. 73, 696 (2017). 10.1107/s2052520617009234 28762979

[49] L. E. Hatcher, M. R. Warren, J. M. Skelton, A. R. Pallipurath, L. K. Saunders, D. R. Allan, P. Hathaway, G. Crevatin, D. Omar, B. H. Williams, B. A. Coulson, C. C. Wilson, and P. R. Raithby, Commun. Chem. 5, 102 (2022). 10.1038/s42004-022-00716-1 36697958 PMC9814726

[50] E. Collet, M.-H. Lemée-Cailleau, M. Buron-Le Cointe, H. Cailleau, M. Wulff, T. Luty, S.-Y. Koshihara, M. Meyer, L. Toupet, P. Rabiller, and S. Techert, Science 300, 612 (2003). 10.1126/science.1082001 12714737

[51] K. A. Deresz, P. Łaski, R. Kamiński, and K. N. Jarzembska, Crystals 11, 1345 (2021). 10.3390/cryst11111345

[52] P. R. Raithby, “Time-resolved single-crystal X-ray crystallography,” in 21st Century Challenges in Chemical Crystallography I. Structure and Bonding, edited by D. M. P. Mingos and P. R. Raithby (Springer, Cham, 2020), Vol. 185.

[53] W. Kabsch, J. Appl. Crystallogr. 26, 795 (1993). 10.1107/s0021889893005588

[54] G. M. Sheldrick, SHELXS-97, A Program for Automatic Solution of Crystal Structures, University of Göttingen, Göttingen, 1997.

[55] G. M. Sheldrick, SHELXL-97, A Program for Crystal Structure Refinement, University of Göttingen, Göttingen, 1997.

[56] P. R. Spackman, M. J. Turner, J. J. McKinnon, S. K. Wolff, D. J. Grimwood, D. Jayatilaka, and M. A. Spackman, J. Appl. Crystallogr. 54, 1006 (2021). 10.1107/s1600576721002910 34188619 PMC8202033

[57] A. D. Becke, J. Chem. Phys. 98, 5648 (1993). 10.1063/1.464913

[58] C. Lee, W. Yang, and R. G. Parr, Phys. Rev. B 37, 785 (1988). 10.1103/physrevb.37.785 9944570

[59] M. J. Frisch, G. W. Trucks, H. B. Schlegel, G. E. Scuseria, M. A. Robb, J. R. Cheeseman, G. Scalmani, V. Barone, G. A. Petersson, H. Nakatsuji, X. Li, M. Caricato, A. V. Marenich, J. Bloino, B. G. Janesko, R. Gomperts, B. Mennucci, H. P. Hratchian, J. V. Ortiz, A. F. Izmaylov, J. L. Sonnenberg, D. Williams-Young, F. Ding, F. Lipparini, F. Egidi, J. Goings, B. Peng, A. Petrone, T. Henderson, D. Ranasinghe, V. G. Zakrzewski, J. Gao, N. Rega, G. Zheng, W. Liang, M. Hada, M. Ehara, K. Toyota, R. Fukuda, J. Hasegawa, M. Ishida, T. Nakajima, Y. Honda, O. Kitao, H. Nakai, T. Vreven, K. Throssell, J. A. Montgomery, Jr., J. E. Peralta, F. Ogliaro, M. J. Bearpark, J. J. Heyd, E. N. Brothers, K. N. Kudin, V. N. Staroverov, T. A. Keith, R. Kobayashi, J. Normand, K. Raghavachari, A. P. Rendell, J. C. Burant, S. S. Iyengar, J. Tomasi, M. Cossi, J. M. Millam, M. Klene, C. Adamo, R. Cammi, J. W. Ochterski, R. L. Martin, K. Morokuma, O. Farkas, J. B. Foresman, and D. J. Fox, Gaussian 16, Revision A.03, Gaussian, Inc., Wallingford CT, 2016.

[60] Y. Zhao and D. G. Truhlar, Theor. Chem. Acc. 120, 215 (2008). 10.1007/s00214-007-0310-x

[61] N. M. O’boyle, A. L. Tenderholt, and K. M. Langer, J. Comput. Chem. 29, 839 (2008). 10.1002/jcc.20823 17849392

[62] R. Dennington, T. A. Keith, and J. M. Millam, GaussView, Version 6, Semichem, Inc., Shawnee Mission, KS, 2016.

[63] T. Lu and F. Chen, J. Comput. Chem. 33, 580 (2012). 10.1002/jcc.22885 22162017

[64] T. Lu, Multiwfn Manual, version 3.6(dev), section 3.21.1 and 3.21.8, available at http://sobereva.com/multiwfn.

[65] G. A. Zhurko, Chemcraft - Graphical Program for Visualization of Quantum Chemistry Computations, Ivanovo, Russia, 2005, https://www.chemcraftprog.com.

[66] P. Coppens, M. Pitak, M. Gembicky, M. Messerschmidt, S. Scheins, J. Benedict, S.-I. Adachi, T. Sato, S. Nozawa, K. Ichiyanagi, M. Chollet, and S.-Y. Koshihara, J. Synchrotron Radiat. 16, 226 (2009). 10.1107/s0909049508040892 19240334 PMC2651764

[67] J. A. Kalinowski, A. Makal, and P. Coppens, J. Appl. Crystallogr. 44, 1182 (2011). 10.1107/s0021889811038143 22199400 PMC3246831

[68] J. A. Kalinowski, B. Fournier, A. Makal, and P. Coppens, J. Synchrotron Radiat. 19, 637 (2012). 10.1107/s0909049512022637 22713901 PMC3380659

[69] R. H. Blessing, Crystallogr. Rev. 1, 3 (1987). 10.1080/08893118708081678

[70] R. H. Blessing, Acta Crystallogr., Sect. A: Found. Adv. 51, 33 (1995). 10.1107/s0108767394005726 7702794

[71] R. H. Blessing, J. Appl. Crystallogr. 30, 421 (1997). 10.1107/s0021889896014628

[72] I. I. Vorontsov and P. Coppens, J. Synchrotron Radiat. 12, 488 (2005). 10.1107/s0909049505014561 15968127

[73] I. Vorontsov, S. Pillet, R. Kamiński, M. S. Schmøkel, and P. Coppens, J. Appl. Crystallogr. 43, 1129 (2010). 10.1107/s0021889810029900

[74] N. J. Hestand and F. C. Spano, Chem. Rev. 118, 7069 (2018). 10.1021/acs.chemrev.7b00581 29664617

[75] N. Mataga, Y. Kaifu, and M. Koizumi, Bull. Chem. Soc. Jpn. 28, 690 (1955). 10.1246/bcsj.28.690

[76] E. Lippert, Z. Naturforsch., A 10, 541 (1955). 10.1515/zna-1955-0707

[77] E. Lippert, Ber. Bunsenges. Phys. Chem. 61, 962 (1957). 10.1002/bbpc.19570610819

[78] X. Zhang, J. Wang, Y. Liu, Y. Hao, F. Yu, D. Li, X. Huang, L. Yu, T. Wang, and H. Hao, J. Phys. Chem. C 125, 6189 (2021). 10.1021/acs.jpcc.0c10536

[79] N. Mataga, T. Okada, H. Masuhara, N. Nakashima, Y. Sakata, and S. Misumi, J. Lumin. 12-13, 159 (1976). 10.1016/0022-2313(76)90075-2

[80] S. Masaki, T. Okada, N. Mataga, Y. Sakata, and S. Misumi, Bull. Chem. Soc. Jpn. 49, 1277 (1976). 10.1246/bcsj.49.1277

[81] P. Chen, R. Duesing, D. K. Graff, and T. J. Meyer, J. Phys. Chem. 95, 5850 (1991). 10.1021/j100168a027

[82] Y. H. Zhao, M. H. Abraham, and A. M. Zissimos, J. Org. Chem. 68, 7368 (2003). 10.1021/jo034808o 12968888

[83] B. K. Hodnett and V. Verma, Processes 7, 272 (2019). 10.3390/pr7050272

[84] M. Sarma and K.-T. Wong, ACS Appl. Mater. Interfaces 10, 19279 (2018). 10.1021/acsami.7b18318 29613766

[85] R. L. Martin, J. Chem. Phys. 118, 4775 (2003). 10.1063/1.1558471

[86] N. Nakashima, N. Mataga, and C. Yamanaka, Int. J. Chem. Kinet. 5, 833 (1973). 10.1002/kin.550050510

[87] K. Nakatani, T. Okada, N. Mataga, F. C. De Schryver, and M. Van der Auweraer, Chem. Phys. Lett. 145, 81 (1988). 10.1016/0009-2614(88)85137-6

[88] S. I. Druzhinin, A. Demeter, and K. A. Zachariasse, Chem. Phys. Lett. 347, 421 (2001). 10.1016/s0009-2614(01)01079-x

[89] P. Coppens, A. Makal, B. Fournier, K. N. Jarzembska, R. Kamiński, K. Basuroy, and E. Trzop, Acta Crystallogr., Sect. B: Struct. Sci., Cryst. Eng. Mater. 73, 23 (2017). 10.1107/s2052520616017558

[90] A. Makal, E. Trzop, J. Sokolow, J. Kalinowski, J. Benedict, and P. Coppens, Acta Crystallogr., Sect. A: Found. Adv. 67, 319 (2011). 10.1107/s0108767311011883 PMC3121236 21694470

[91] M. D. Carducci, M. R. Pressprich, and P. Coppens, J. Am. Chem. Soc. 119, 2669 (1997). 10.1021/ja9625743

[92] B. Fournier and P. Coppens, Acta Crystallogr., Sect. A: Found. Adv. 70, 291 (2014). 10.1107/s2053273314006305 24815977 PMC4011010

[93] M. S. Schmøkel, R. Kamiński, J. B. Benedict, and P. Coppens, Acta Crystallogr., Sect. A: Found. Adv. 66, 632 (2010). 10.1107/S0108767310029429 20962370

[94] E. D. Glendening, C. R. Landis, and F. Weinhold, Wiley Interdiscip. Rev.: Comput. Mol. Sci. 2, 1 (2012). 10.1002/wcms.51

[95] A. E. Reed, L. A. Curtiss, and F. Weinhold, Chem. Rev. 88, 899 (1988). 10.1021/cr00088a005

[96] K. Basuroy, J. d. J. Velazquez-Garcia, D. Storozhuk, D. J. Gosztola, S. T. Veedu, and S. Techert, J. Chem. Phys. 155, 234304 (2021). 10.1063/5.0072785 34937351

[97] K. Pettersson, J. Wiberg, T. Ljungdahl, J. Mårtensson, and B. Albinsson, J. Phys. Chem. A 110, 319 (2006). 10.1021/jp054420s 16392871

